# *TYMP* Variants Result in Late-Onset Mitochondrial Myopathy With Altered Muscle Mitochondrial DNA Homeostasis

**DOI:** 10.3389/fgene.2020.00860

**Published:** 2020-08-05

**Authors:** Dario Ronchi, Leonardo Caporali, Giulia Francesca Manenti, Megi Meneri, Susan Mohamed, Andreina Bordoni, Francesca Tagliavini, Manuela Contin, Daniela Piga, Monica Sciacco, Cristina Saetti, Valerio Carelli, Giacomo Pietro Comi

**Affiliations:** ^1^IRCCS Fondazione Ca’ Granda Ospedale Maggiore Policlinico, Neurology Unit, Milan, Italy; ^2^Dino Ferrari Center, Department of Pathophysiology and Transplantation, University of Milan, Milan, Italy; ^3^IRCCS Istituto delle Scienze Neurologiche di Bologna, Bologna, Italy; ^4^Dipartimento di Scienze Biomediche e Neuromotorie (DIBINEM), Università di Bologna, Bologna, Italy; ^5^IRCCS Fondazione Ca’ Granda Ospedale Maggiore Policlinico, Neuromuscular and Rare Disease Unit, Milan, Italy

**Keywords:** mitochondrial myopathy, mitochondrial DNA instability, *TYMP*, mitochondrial neurogatrointestinal encephalomyopathy, mitochondrial DNA replication

## Abstract

Biallelic *TYMP* variants result in the mitochondrial neurogastrointestinal encephalomyopathy (MNGIE), a juvenile-onset disorder with progressive course and fatal outcome. Milder late-onset (>40 years) form has been rarely described. Gene panel sequencing in a cohort of 60 patients featuring muscle accumulation of mitochondrial DNA (mtDNA) deletions detected *TYMP* defects in three subjects (5%), two of them with symptom onset in the fifth decade. One of the patients only displayed ptosis and ophthalmoparesis. Biochemical and molecular studies supported the diagnosis. Screening of *TYMP* is recommended in adult patients with muscle mtDNA instability, even in the absence of cardinal MNGIE features.

## Introduction

Thymidine phosphorylase (TP) is a cytosolic enzyme that catalyzes the conversion of pyrimidine nucleosides thymidine and deoxyuridine into the corresponding bases by releasing 2-deoxy-1-phosphate ribose. Loss of TP activity results in the marked accumulation of its substrates, reaching toxic levels in plasma and other tissues (Spinazzola et al., 2002). Mitochondrial DNA (mtDNA) lacks an effective mismatch repair system (Bohr and Anson, 1999) and is particularly susceptible to dNTP imbalance due to TP deficiency (Hirano et al., 2012). As a consequence, quantitative and qualitative mtDNA changes might be found in affected tissues, including muscle (Papadimitriou et al., 1998).

Loss of function changes in *TYMP*, the gene ending TP, have been associated with a peculiar clinical presentation known as mitochondrial neurogastrointestinal encephalomyopathy (MNGIE, MIM 603041) featuring the following clinical hallmarks: extraocular muscle weakness, gastrointestinal (GI) dysmotility, cachexia, sensorimotor peripheral neuropathy, and leukoencephalopathy (Nishino et al., 1999). The vast majority of cases (>95%) exhibit an onset before the age of 20 years with a progressive course leading to a fatal outcome within the fourth decade (classical form) (Garone et al., 2011). Four late-onset (beyond 40 years of age) MNGIE cases have been described: they presented mild symptoms (including GI disturbances) and slow progression (Martí et al., 2005; Massa et al., 2009). The observation that residual TP levels are higher in late-onset compared with juvenile patients has prompted the investigation of preclinical therapeutic approaches, which are progressing toward clinical use (Yadak et al., 2017).

We applied a next-generation sequencing panel to increase the diagnostic rate in a group of adult patients featuring muscle mtDNA deletions, and we disclosed *TYMP* variants in three subjects, two of them manifesting mainly isolated mitochondrial myopathy. Biochemical and molecular studies supported the diagnosis.

## Methods

The study was approved by the institutional review board of the IRCCS Fondazione Ca’ Granda Ospedale Maggiore Policlinico. The patients provided written informed consent for all aspects of the study including the publication of any potentially identifiable data included in this article.

Genes involved in mtDNA maintenance disorders were investigated by a panel including 24 genes associated with mtDNA instability (Supplementary Table 1) starting from blood-derived DNA. The library was generated using a 250-bp amplicon-based approach (TruSeq Custom Amplicon, Illumina) and sequenced on MiSeq instrument (Illumina). Reads were aligned to the human genome (assembly hg19), and the identified variants were annotated (ANNOVAR) and filtered, focusing on rare variants (≤0.5% in public databases), causing changes potentially damaging for the protein function (CADD and DANN).

Sanger sequencing validation was performed for the variants detected in *TYMP* (NM_001113755).

Muscle mtDNA was assayed by Southern blot and long-PCR analysis as previously described (Ronchi et al., 2012). MtDNA content and integrity were investigated by relative quantification on an ABI 7500 Real Time PCR System (Ronchi et al., 2012). Determinations were performed in triplicates. *MT-CYTB*, *APP*, *MT-ND4*, and *MT-ND1* regions were simultaneously probed by fluorescent TaqMan assays. MtDNA sequencing was performed by Sanger method.

The assessment of residual leukocytes TP activity was performed as previously described (Nishino et al., 1999). TP (Cell Signaling, BK4307S, 1:1,000) levels were assessed by sodium dodecyl sulfate–polyacrylamide gel electrophoresis (SDS-PAGE) in leukocytes of Patient 1 and controls and normalized to ACTIN (Sigma A2066, 1:1,500) levels. Thymidine and deoxyuridine plasma levels were measured on high-performance liquid chromatography–ultraviolet (HPLC-UV), as previously described (Mohamed et al., 2014).

## Results

We considered a cohort of 60 adult patients, collected at Ospedale Maggiore Policlinico in Milan in the last 30 years, and presenting a clinical suspect of mitochondrial disease supported by muscle histochemical findings (presence of cytochrome *c* oxidase negative and/or ragged red fibers) and featuring the accumulation of multiple mtDNA deletions in muscle (Southern blot or long-range PCR analysis). Symptom onset ranged from 16 to 83 years of age (mean age at onset = 54 ± 19 years).

**FIGURE 1:**
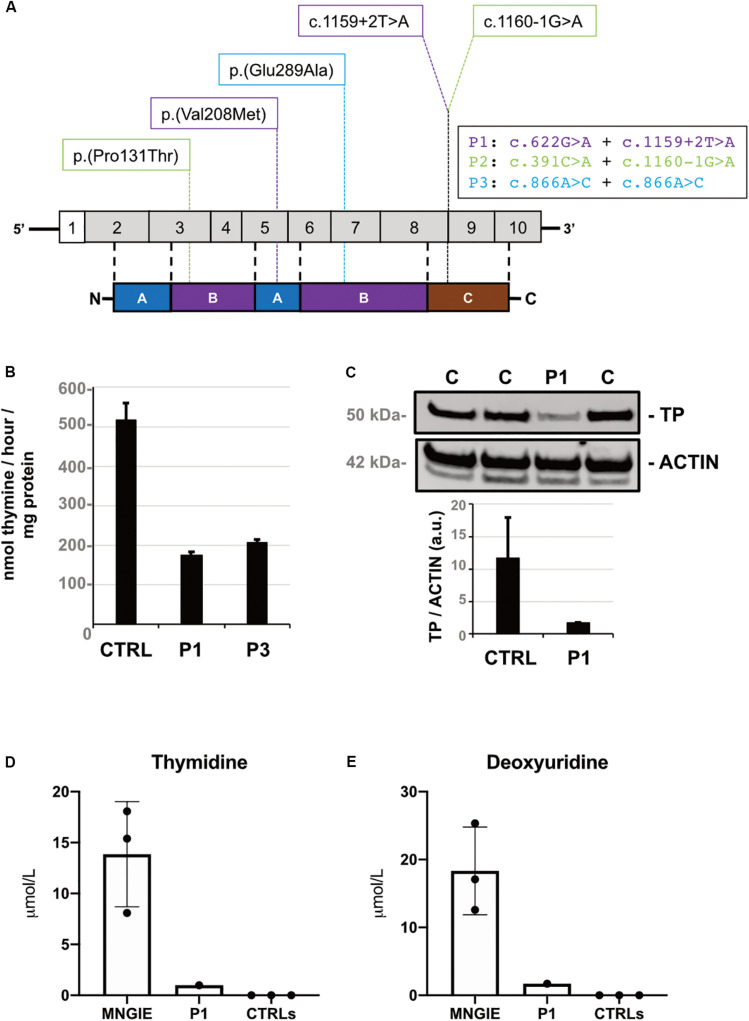
Molecular and biochemical findings. **(A)** Diagram of *TYMP* and its encoded protein thymidine phosphorylase (TP) showing variants identified by gene panel sequencing. **(B)** TP activity levels (expressed as nanomoles of thymine per hour per milligram of protein) in leukocytes of Patient 1, Patient 3, and normal controls (*n* = 6). **(C)** Western blot and densitometric analysis of residual TP protein levels in leucocytes of Patient 1 compared with control samples (*n* = 3). TP proteins are normalized to ACTIN (arbitrary units). **(D)** Thymidine and **(E)** deoxyuridine plasma levels in Patient 1 compared with controls (*n* = 7) and MNGIE patients (*n* = 3). Data are expressed as μmol per Liter and presented as scatter plot with bar for mean and standard deviation.

After ruling out genes encoding for proteins involved in mtDNA replication and repair (Ahmed et al., 2015), we investigated additional genes linked with mtDNA disturbances by gene panel sequencing. We detected suspicious *TYMP* variants in three subjects (5%). Clinical, instrumental, and molecular findings are summarized in Table 1. Patient 1 was compound heterozygous for the c.622G > A p.(Val208Met) substitution (allele frequencies: gnomAD = 3.18E-04, Topmed = 1.75E-04) and the splice-site change c.1159 + 2T > A (Figure 1A). Patient 2 was compound heterozygous for the novel c.391C > A p.(Pro131Thr) substitution (Topmed = 7.96E-06) and the splice-site variant c.1160-1G > A (Figure 1A). Patient 3 harbored the homozygous c.866A > C p.(Glu289Ala) missense change (Topmed = 7.17E-05, Figure 1A). DNA from relatives was not available for segregation studies. Missense changes were predicted to be deleterious by Polyphen^1^, SIFT^2^, CADD^3^, MutPred^4^, Mutation Assessor^5^, and Mutation Taster^6^. Splice-site variants were predicted to alter canonical splicing by ASSP^7^, NetGene2^8^, and Human Splicing Finder^9^ tools. With the exception of the novel change c.391C > A, all the other variants detected have been previously reported in MNGIE patients (Garone et al., 2011): in particular, nucleotide changes c.622G > A and c.1160-1G > A have been reported in three of four late-onset MNGIE cases (Martí et al., 2005; Massa et al., 2009), suggesting a genotype–phenotype correlation.

**TABLE 1:** Clinical, instrumental, and molecular findings of *TYMP* patients described in this study (P1–P3) and previously reported late-onset MNGIE patients.

Patient	Gender, country	Age (years)	Age at onset (years)	Clinical features	Brain MRI	EMG	Muscle biopsy	*TYMP* allele 1	*TYMP* allele 2	TP activity leukocytes	MtDNA (muscle)
P1, this study	M, Italian	67	48	Ptosis, ophthalmoparesis	Normal	n.p.	COX- (3.33%) RRF (5%)	c.622G > A p.(Val208Met)	c.1159 + 2T > A	174 (34%)	MtDNA dels (SB, lrPCR)
P2, this study	M, Italian	73	43	Ptosis, ophthalmoparesis, muscle weakness, peripheral neuropathy, dysphagia, hypoacusis, psychiatric symptoms, cardiac symptoms, gastrointestinal dysmotility, diabetes, dysthyroidism, Parkinsonism	n.p.	N	RRF (0.5%)	c.391C > A p.(Pro131Thr)	c.1160-1G > A	n.p.	MtDNA dels (SB, lrPCR)
P3, this study	F, Greek	34	18	Muscle weakness, ptosis, ophthalmoparesis and hypoacusis	n.p.	N	COX- RRF	c.866A > C p.(Glu289Ala)	c.866A > C p.(Glu289Ala)	208 (40%)	MtDNA dels (lrPCR)
Martí et al. (2005) (Pat. 1)	F, Anglo-American	61	42	Ptosis, ophthalmoparesis, muscle weakness, peripheral neuropathy, optic atrophy, hearing loss, gastrointestinal disturbances	Leukoencephalopathy	n.p.	n.p.	c.622G > A p.(Val208Met)	c.931G > C p.(Gly311Ser)	105 (16%)	n.a.
Martí et al. (2005) (Pat. 2)	F, Dutch	55	40	Ptosis, ophthalmoparesis, muscle weakness, gastrointestinal disturbances	Leukoencephalopathy	N	COX-	c.622G > A p.(Val208Met)	c.605G > C p.(Arg202Thr)	89 (15%)	n.a.
Martí et al. (2005) (Pat. 3)	M, Anglo-American	57	52	Ptosis, ophthalmoparesis, muscle weakness, peripheral neuropathy, hearing loss, cardiac symptoms, gastrointestinal dysmotility, diabetes	Leukoencephalopathy	n.p.	COX- RRF	c.457G > A p.(Gly153Ser)	c.854T > C p.(Leu285Pro)	88 (13%)	n.a.
Massa et al. (2009)	F, Italian	67	53	Ptosis, ophthalmoparesis, hearing loss, gastrointestinal disturbances	Leukoencephalopathy	M	COX- RRF/SDH+	c.1135G > A p.(Glu379Lys)	c.1160-1G > A	132 (18%)	MtDNA dep (qPCR, 43%)

*EMG, electromyography; N, neurogenic; M, myopathic; n.p., not performed; COX-, cytochrome c oxidase negative fibers; SDH+, succinate dehydrogenase positive fibers; RRF, ragged red fibers; mtDNA dels, mitochondrial DNA multiple deletions; mtDNA dep, mitochondrial DNA depletion; SB, Southern blot; qPCR, quantitative PCR; lrPCR, long-range PCR.*

TP activity was found decreased in leukocytes of Patient 1 (174.4 nmol/mg/h) and Patient 3 (208.0 nmol/mg/h) representing, respectively, 34 and 40% of in-house control values (515.6 ± 43.6 nmol/mg/h) (Figure 1B). Additional investigations in Patient 1 showed reduced TP protein content (about 15% of controls, Figure 1C) and increased plasma accumulation of thymidine (1.0 μmol/L) and deoxyuridine (1.7 μmol/L), undetectable in healthy controls (Figures 1D,E).

**FIGURE 2:**
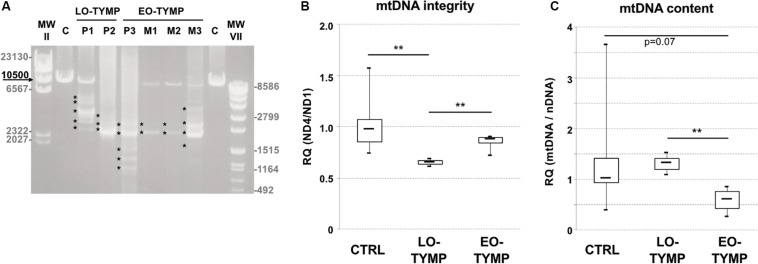
Mitochondrial DNA studies in *TYMP* patients. **(A)** Long-range PCR analysis of mitochondrial DNA obtained from late-onset *TYMP* patients’ muscle biopsies (P1–P2, LO-TYMP), early onset *TYMP* patients (P3 and M1–M3, EO-TYMP), and healthy donors **(C)**. Asterisks indicate multiple bands corresponding to multiple mtDNA deletions. Black arrow indicates the expected size of wild-type PCR amplicon (10.5 kB: FOR5635-RC16135). The sizes of the bands of the ladders (DNA Molecular Weight Marker II and VII, Roche) are indicated. **(B)** Box plot showing muscle mtDNA integrity in late-onset *TYMP* patients (LO-TYMP, *n* = 3) compared with early onset patients (EO-TYMP, *n* = 4) and healthy controls (*n* = 27). Solid bars represent median values. The ND4/ND1 ratio indicates the amount of normal (non-deleted) molecules with respect to the total number of mitochondrial genomes amplified by quantitative PCR (**p < 0.05, *t*-test). **(C)** Box plot showing muscle mtDNA content in late-onset *TYMP* patients (LO-TYMP, *n* = 3) compared with early onset patients (EO-TYMP, *n* = 4) and healthy controls (*n* = 27). Solid bars represent median values. MtDNA quantification, normalized to nuclear DNA (nDNA) content, was performed by quantitative PCR (**p < 0.05, *t*-test).

We evaluated mtDNA content and integrity in muscle specimens of these patients and of three siblings presenting classical MNGIE phenotype, previously reported by our group (Papadimitriou et al., 1998). Long-range PCR analysis showed the presence of multiple mtDNA deletions in all *TYMP*-mutated subjects with higher accumulation in late-onset patients (Figure 2A). This finding was confirmed by quantitative PCR studies (Figure 2B). Decreased mtDNA content was observed in muscle of early onset but not late-onset patients (Figure 2C).

Full mtDNA sequencing performed in muscle and leukocytes of Patient 1 (Supplementary Table 2) and Patient 2 (Supplementary Table 3) revealed previously reported nucleotide substitutions without an obvious pathogenetic role. All the changes observed were found homoplasmic in matched muscle and blood samples with the only exception of the control region age-related A189G variant (Wang et al., 2001), which was observed in muscle but not in leukocytes of Patient 2.

## Discussion

MtDNA instability affects every stage of life. In infants, severe reduction of mtDNA content in affected tissues result in early onset multisystemic presentations (mtDNA depletion syndrome). Progressive external ophthalmoplegia (PEO) and limb weakness are predominant symptoms in adult presentations reflecting the detrimental accumulation of mtDNA deletions in muscles. However, the involvement of other tissues and organs is not uncommon (Viscomi and Zeviani, 2017; Del Dotto et al., 2020).

Recent advancements in the genetics of mitochondrial disorders highlight the overlap between early and late-onset presentations: genes associated with pediatric presentations are also mutated in adult patients or vice versa. Therefore, molecular defects in the same gene might result in a spectrum of phenotypes ranging from early onset life-threatening conditions to presentations with delayed onset and mild progressive course (Viscomi and Zeviani, 2017).

More than 120 MNGIE patients have been described presenting an average age at onset of 17.9 years and a progressive course resulting fatal within two decades (Hirano, 2016). Beside ptosis and PEO, cachexia and GI dysmotility are frequently observed in this presentation. In a few cases, typical MNGIE symptoms occurred after 40 years of age with mild progression (Table 1). The co-occurrence of multiple deletions, depletion, and somatic mtDNA point mutations was often described in muscle of MNGIE patients, as a consequence of mitochondrial dNTP imbalance due to TP deficiency (Hirano et al., 1994; Nishigaki et al., 2003).

Juvenile onset and peculiar clinical features have directed molecular testing of *TYMP* in selected patients in the last years.

Next generation sequencing allows the simultaneous analyses of multiple genes involved in mtDNA maintenance, preventing the need of prioritization of genes to be tested on the basis of available clinical and instrumental findings.

By applying this strategy to 60 undiagnosed adult patients with PEO and/or limb weakness and muscle mtDNA instability, we detected recessive *TYMP* defects in three novel patients. Accurate examination of clinical records in one of them (Patient 2) revealed a complex phenotype including GI disturbances, diabetes, dysthyroidism, and extrapyramidal signs (by the age of 66 years). Interestingly, parkinsonism has been described in several adult presentations with altered mtDNA stability (Ronchi et al., 2018).

Patients 1 and 3 displayed predominant muscle phenotype, indistinguishable from adult patients presenting mitochondrial myopathy with mtDNA deletions. Despite identical genotype with typical MNGIE cases (Nishino et al., 1999; Slama et al., 2005; Amiot et al., 2009; Bakker et al., 2010; Finkenstedt et al., 2013), Patient 3 did not disclose GI disturbances. Beside ocular symptoms (ptosis and external ophthalmoplegia), she displayed peripheral neuropathy and hearing loss, which are reported as starting symptoms in less than 15% of MNGIE patients (Garone et al., 2011). Unexpectedly high levels (40% of our controls) of residual blood TP activity might explain this difference.

Our findings expand the spectrum of clinical presentations due to *TYMP* variants and suggest extending the analysis of this gene even in those cases, which do not manifest the cardinal features of MNGIE.

TP activity levels were found reduced in leukocytes of Patients 1 (34% of our controls, respectively). Even considering reference control values from other laboratories (634 ± 17 nmol/h/mg) (Martí et al., 2004), residual activities of these subjects are higher than those of other late-onset MNGIE patients (16% of controls). Accordingly, plasma levels of thymidine (1.0 μmol/L) and deoxyuridine (1.7 μmol/L) assayed in Patient 1 were lower compared with mean values observed in late-onset MNGIE cases (thymidine: 1.4 ± 0.6 and deoxyuridine 2.3 ± 1.7 μmol/L) but still higher with respect to control (undetectable) (Li et al., 2019).

The co-occurrence of multiple deletions, depletion, and somatic mtDNA point substitutions was often described in muscle of MNGIE patients. We can speculate that massive plasma accumulation of thymidine in MNGIE patients results in consistent mtDNA depletion in muscle whereas moderate levels of thymidine observed in late-onset TYMP-patients prime the age-dependent accumulation of mtDNA deletions but are not sufficient to significantly affect mtDNA content and polymerase fidelity. Indeed, we did not observe the accumulation of somatic mtDNA point substitutions in available tissues from Patient 1, whereas Patient 2’s muscle harbored the A189G variant, previously identified as a muscle-specific nucleotide change accumulating with aging (Wang et al., 2001). We can speculate that TP deficiency might be related with the occurrence of this somatic variant in Patient 2, although this A–G change does not match the features of mtDNA sequence abnormalities detected in MNGIE patients (Nishigaki et al., 2003). We have previously showed that control-region variants are increased in muscles of patients displaying mtDNA instability, including two early onset MNGIE cases where the A189G was found with a low level (<5%) of heteroplasmy (Del Bo et al., 2003). Other authors suggested that the frequency of A189G substitution positively correlates with age but does not differ between mitochondrial patients and healthy subjects (da Costa et al., 2007).

It was suggested that 26–35% of residual TP activity is sufficient to prevent disease onset. Our findings argue against this conclusion and invite to establish clinical surveillance for subjects harboring hypomorphic *TYMP* alleles.

Finally, the identification of adult *TYMP* patients will permit their enrollment in ongoing clinical trials.

## Data Availability Statement

All datasets generated for this study are included in the article/Supplementary Material, further inquiries can be directed to the corresponding author.

## Ethics Statement

The studies involving human participants were reviewed and approved by the Institutional review board of the IRCCS Fondazione Ca’ Granda Ospedale Maggiore Policlinico. The patients/participants provided their written informed consent to participate in this study. Written informed consent was obtained from the individual(s) for the publication of any potentially identifiable images or data included in this article.

## Author Contributions

MM and CS reviewed patients’ clinical records and performed the clinical assessment. DR, LC, FT, and GM performed the genetic and molecular studies. AB, DP, SM, and MC performed the biochemical studies. MS, VC, and GC revised the manuscript. DR and LC were responsible for drafting the manuscript and preparing figures. All authors contributed to the article and approved the submitted version.

## Conflict of Interest

The authors declare that the research was conducted in the absence of any commercial or financial relationships that could be construed as a potential conflict of interest.

## Footnotes

1 http://genetics.bwh.harvard.edu/pph2/

2 https://sift.bii.a-star.edu.sg

3 https://cadd.gs.washington.edu

4 http://mutpred1.mutdb.org

5 http://mutationassessor.org/r3/

6 http://www.mutationtaster.org

7 http://wangcomputing.com/assp/

8 http://www.cbs.dtu.dk/services/NetGene2/

9 http://umd.be/Redirect.html
