# Supplementary material for: TYMP Variants Result in Late-Onset Mitochondrial Myopathy With Altered Muscle Mitochondrial DNA Homeostasis
Source: Front Genet. 2020 Aug 5;11:860. doi: 10.3389/fgene.2020.00860 (PMC7419576; doi:10.3389/fgene.2020.00860)
Supplement: Supplementary file 1 [file Data_Sheet_1.docx]

**TYMP mutations result in late onset mitochondrial myopathy**

**with altered muscle mtDNA homeostasis**

**Supplementary Information**

**Supplementary Table 1**: genes included in the panel used for NGS sequencing

**Supplementary Table 2**: mitochondrial DNA sequence of Patient 1

**Supplementary Table 3:** mitochondrial DNA sequence of Patient 2

| **Gene** | **Inheritance** | **OMIM** | **Locus** | **Refseq** | **Reference (PMID)** |
| --- | --- | --- | --- | --- | --- |
| AFG3L2 | AD | *604581, #610246 | 18p11.21 | NM_006796.3 | 25420100 |
| CHCHD10 | AD | *615903, #615911 | 22q11.23 | NM_001301339.2 | 24934289 |
| DGUOK | Ar | *601465, #617070 | 2p13.1 | NM_080916.3 | 23043144 |
| DNA2 | AD | *601810, # 615156 | 10q21.3 | NM_001080449.3 | 23352259 |
| DNM1L | AD, Ar | *603850 | 12p11.21 | NM_012062.5 | - |
| FBXL4 | Ar | *605654, #615471 | 6q16.1-q16.2 | NM_012160.4 | - |
| GFER | Ar | *600924, #613076 | 16p13.3 | NM_005262.3 | 19409522 |
| MFN1 | unknown | *608506 | 3q26.33 | NM_001206614.1 | - |
| MFN2 | AD | *608507 | 1p36.22 | NM_014874.4 | 22189565 |
| MGME1 | Ar | *615076 | 20p11.23 | NM_001310338.2 | 23313956 |
| MPV17 | Ar | *137960 | 2p23.3 | NM_002437.5 | 22508010 |
| OPA1 | AD | *605290, #125250 | 3q29 | NM_130837.3 | 18158317 |
| POLG | AD, Ar | *174763, #157640, #258450 | 15q26.1 | NM_002693.3 | 11431686 |
| POLG2 | AD | *604983, #610131 | 17q23.3 | NM_007215.4 | 16685652 |
| RNASEH1 | Ar | *604123, #616479 | 2p25.3 | NM_002936.6 | 26094573 |
| RRM2B | Ar | *604712, #613077 | 8q22.3 | NM_015713.5 | 21646632 |
| SLC25A4 | AD | *103220, #609283 | 4q35.1 | NM_001151.4 | 10926541 |
| SPG7 | Ar | *602783 | 16q24.3 | NM_001363850.1 | 18200586 |
| SSBP1 | AD | *600439 | 7q34 | NM_001256510.1 | - |
| SUCLA2 | Ar | *603921, #612073 | 13q14.2 | NM_003850.3 | - |
| SUCLG1 | Ar | *611224, #245400 | 2p11.2 | NM_003849.4 | - |
| TK2 | Ar | *188250, #617069 | 16q21 | NM_004614.5 | 21937588 |
| TWNK | AD | *606075, #609286 | 10q24.31 | NM_021830.5 | 7719341 |
| TYMP | Ar | *131222, #603041 | 22q13.33 | NM_001953.5 | 9924029 |

**Supplementary Table 1 Genes associated with mtDNA instability leading to multiple deletions or candidate genes.**

For each gene associated, the first report with documented mtDNA multiple deletions associated with pathogenic mutation are reported as PubMed unique identifier (PMID). AD=Autosomal Dominant, Ar=Autosomal recessive.

| **Position** | **rCRS NT** | **Alt Seq** | **Locus** | **Gene Bank Frequency** | **Leukocytes** |
| --- | --- | --- | --- | --- | --- |
| 73 | A | G | Control Region | 75.91% | + |
| 263 | A | G | Control Region | 94.86% | + |
| 285 | C | T | Control Region | 0.4% | + |
| 385 | A | G | Control Region | 0.37% | + |
| 750 | A | G | Control Region | 98.3% | + |
| 1438 | A | G | 12S | 94.82% | + |
| 2706 | A | G | 16S | 78.94% | + |
| 3158 | A | AT | 16S | 0.10% | + |
| 3591 | G | A | ND1 | 0.84% | + |
| 4769 | A | G | ND2 | 97.6% | + |
| 4991 | G | A | ND2 | 0.53% | + |
| 5567 | T | C | tRNA-W | 0.10% | + |
| 6026 | G | A | COI | 1.77% | + |
| 7028 | C | T | COI | 80.76% | + |
| 7581 | T | C | tRNA-D | 0.46% | + |
| 8860 | A | G | ATPase6 | 98.47% | + |
| 9389 | A | G | COIII | 0.10% | + |
| 9575 | G | A | COIII | 0.68% | + |
| 11467 | A | G | ND4 | 12.55% | + |
| 11719 | G | A | ND4 | 77.40% | + |
| 12308 | A | G | tRNA-L(CUN) | 12.51% | + |
| 12372 | G | A | ND5 | 13.54% | + |
| 12879 | T | C | ND5 | 0.65% | + |
| 13104 | A | G | ND5 | 0.61% | + |
| 13422 | A | G | ND5 | 0.22% | + |
| 14070 | A | G | ND5 | 0.43% | + |
| 14364 | G | A | ND6 | 0.81% | + |
| 14766 | C | T | Cytb | 76.88% | + |
| 15148 | G | A | Cytb | 0.67% | + |
| 15326 | A | G | Cytb | 98.65% | + |
| 15954 | A | C | Control Region | 0.41% | + |
| 16172 | T | C | Control Region | 7.46% | + |
| 16183 | A | C | Control Region | 13.41% | + |
| 16189 | T | C | Control Region | 25.60% | + |
| 16249 | T | C | Control Region | 1.99% | + |

**Supplementary Table 2 Mitochondrial DNA sequence of Patient 1 (haplotype U1a1a1).**

Position indicates nucleotide position in mtDNA. rCRS indicates the nucleotide included in the Revised Cambridge Reference Sequence of human mitochondrial DNA (NC_012920). Alt Seq indicate the nucleotide position called by sequencing muscle-extracted mitochondrial DNA in the patient. Leukocytes indicate whether the variant was present (+) or absent (-) in leukocytes obtained from Patient 2.

| **Position** | **rCRS NT** | **Alt Seq** | **Locus** | **Gene Bank Frequency** | **Leukocytes** |
| --- | --- | --- | --- | --- | --- |
| 189 | A | G | Control Region | 5.51% | - |
| 750 | A | G | Control Region | 98.3% | + |
| 263 | A | G | Control Region | 94.86% | + |
| 1438 | A | G | 12S | 94.82% | + |
| 3010 | G | A | 16S | 14.36% | + |
| 4769 | A | G | ND2 | 97.57% | + |
| 8775 | C | T | ATPase6 | 0.01% | + |
| 8860 | A | G | ATPase6 | 98.47% | + |
| 12501 | G | A | ND5 | 2.60% | + |
| 12678 | T | C | ND5 | 0.06% | + |
| 15326 | A | G | Cytb | 98.65% | + |
| 16519 | T | C | Control Region | 62.82% | + |

**Supplementary Table 3 Mitochondrial DNA sequence of Patient 2 (Haplotype H1).**

Position indicates nucleotide position in mtDNA. rCRS indicates the nucleotide included in the Revised Cambridge Reference Sequence of human mitochondrial DNA (NC_012920). Alt Seq indicate the nucleotide position called by sequencing muscle-extracted mitochondrial DNA in the patient. Leukocytes indicate whether the variant was present (+) or absent (-) in leukocytes obtained from Patient 2.
